# Prolactin levels and female sexual distress: associations with sexual knowledge and behaviors

**DOI:** 10.1093/sexmed/qfag052

**Published:** 2026-07-02

**Authors:** Melih Mustafa Sedef, Bayram Özağaç, Mustafa Şanlı, Arda B Karaduman, Erhan H Cömert

**Affiliations:** Department of Psychiatry, Sungurlu State Hospital, 19300, Çorum, Türkiye; Department of Psychiatry, Etlik City Hospital, 06170, Ankara, Türkiye; Department of Obstetrics and Gynecology, Sungurlu State Hospital, 19300, Çorum, Türkiye; Department of Obstetrics and Gynecology, Tokat Gaziosmanpaşa University Hospital, 60030, Tokat, Türkiye; Department of Obstetrics and Gynecology, Kars Harakani State Hospital, 36200, Kars, Türkiye; Private Practice Obstetrics and Gynecology, 34367, Istanbul, Türkiye

**Keywords:** prolactin, sexual distress, FSDS-R, sexual knowledge, female sexual dysfunction, cross-sectional study, hyperprolactinemia

## Abstract

**Introduction:**

Prolactin (PRL) abnormalities can negatively affect sexual function, yet their relationship with subjective female sexual distress remains insufficiently characterized. The primary aim of this study was to examine the association between serum PRL levels and female sexual distress. As a secondary exploratory aim, we assessed whether sexual knowledge and selected sexual health behaviors were independently associated with sexual distress.

**Methods:**

This cross-sectional study included 200 sexually active women aged ≥18 years attending a gynecology outpatient clinic. Participants completed the Female Sexual Distress Scale–Revised (FSDS-R) and a structured questionnaire evaluating sexual knowledge and sexual health behaviors, including Kegel exercise knowledge and practice. Morning fasting serum PRL levels were measured between 08:00 and 10:00. Participants were categorized as having normal serum PRL (≤25 ng/mL) or elevated serum PRL (>25 ng/mL). Secondary exploratory analyses stratified PRL into sample-based quartiles. Between-group comparisons and analysis of covariance models were used to examine associations with FSDS-R total scores.

**Results:**

Sixty-five participants (32.5%) had elevated PRL. Women with elevated PRL had higher FSDS-R total scores than those with normal PRL (26.1 ± 12.5 vs 22.2 ± 9.4; *P* = .029). In adjusted analyses, elevated PRL remained independently associated with higher sexual distress (Model 1: F = 5.40, *P* = .021; Model 2: F = 5.17, *P* = .024). In secondary exploratory analyses, FSDS-R total scores differed significantly across PRL quartiles (Kruskal–Wallis H[3] = 8.833, *P* = .032), and PRL quartile remained significant in the fully adjusted model (F[3189] = 4.579, *P* = .004). Higher Sexual Knowledge Index scores were independently associated with lower FSDS-R total scores (F = 4.32, *P* = .039), whereas the PRL status × Sexual Knowledge interaction was not significant.

**Discussion:**

Elevated PRL was independently associated with greater female sexual distress, and exploratory quartile analyses supported higher distress in the upper PRL quartile without evidence that the lowest PRL quartile was associated with worse distress. Higher sexual knowledge was independently associated with lower distress. These findings support a biopsychosocial framework in which hormonal and cognitive-educational factors contribute to women’s subjective sexual well-being. Key limitations include the cross-sectional design, single PRL measurement, absence of routine macroprolactin screening, lack of menstrual-cycle-phase standardization, and use of an exploratory Sexual Knowledge Index.

## Introduction

Female sexual health is influenced by a combination of biological, psychological, relational, and sociocultural elements.^1^^,^^2^ According to the Diagnostic and Statistical Manual of Mental Disorders (DSM-V), female sexual dysfunction is categorized as female sexual interest/arousal disorder, female orgasmic disorder, and genito-pelvic pain/penetration disorder. Diagnostic systems require not only the presence of symptoms, but also clinically significant distress and persistence (typically ≥6 months) to distinguish temporary difficulties from disorder.^3^^,^^4^

Not all sexual complaints result in dissatisfaction or distress. Sexual distress is defined as negative personal appraisal and emotional responses (eg, anxiety, shame, sadness, worry) about one’s sexual experiences or perceived problems.^5^^,^^6^ It has clinical importance beyond sexual symptoms and is closely associated with seeking help and overall quality of life.^6^^,^^7^ Given that distress is subjective, consensus documents highlight the importance of using validated patient-reported outcome measures to assess sexual symptoms, distress, and functional impact.^8^^,^^9^

Prolactin (PRL) is a pleiotropic neuroendocrine hormone that influences sexual function through endocrine and neurobehavioral mechanisms. PRL acts on the hypothalamic–pituitary–gonadal axis by inhibiting the release of gonadotropin-releasing hormone and the production of sex steroids when its levels are elevated. Furthermore, PRL is linked to dopaminergic pathways that play a role in sexual motivation and reward processing.^10^^,^^11^ Clinically overt hyperprolactinemia consistently contributes to sexual dysfunction, particularly reducing sexual desire and impairing arousal.^12–14^

The relationship between mildly elevated PRL levels and subjective sexual distress remains unclear. Studies using instruments like the FSDS-R often focus on women with overt hyperprolactinemia or include small subsamples of affected individuals.^15^ While mild PRL elevations have been linked to objective sexual dysfunction indicators, evidence that such elevations independently contribute to clinical sexual distress is limited. Available data emphasizes sexual function scores rather than distress as a distinct construct.

Sexual knowledge and education may influence how women understand and interpret sexual experiences.^16^ Limited knowledge of genital anatomy and sexual response may contribute to misconceptions and anxiety, potentially increasing distress even with mild physiological impairment. Studies show that increasing sexual knowledge and self-efficacy improves function and reduces distress. However, increased awareness without skill development or support could heighten sensitivity to unmet expectations.^17^^,^^18^ There is limited empirical research exploring sexual knowledge in conjunction with biological factors like PRL.

Against this background, the primary objective of the present study was to compare sexual distress between women with elevated versus normal serum PRL levels. As secondary exploratory objectives, we examined whether sexual knowledge and selected sexual health behaviors, including Kegel exercise knowledge and practice, were independently associated with sexual distress after adjustment for PRL status and clinical covariates. These secondary variables were included within a biopsychosocial framework because sexual knowledge may influence distress through cognitive appraisal, sexual misconceptions, and sexual self-efficacy, whereas pelvic floor-related knowledge and behaviors may influence sexual function and indirectly contribute to sexual distress. Therefore, we tested the following refutable research question: Are elevated serum PRL levels associated with higher female sexual distress, and are sexual knowledge and selected sexual health behaviors independently associated with sexual distress after adjustment for relevant clinical covariates?

## Materials and methods

### Study design and setting

This cross-sectional study was conducted at a gynecology and obstetrics outpatient clinic between May 2025 and January 2026. The study protocol was approved by the relevant Institutional Review Board/Ethics Committee, with full approval details provided in the title page for editorial review, and was conducted in accordance with the Declaration of Helsinki. All participants provided written informed consent before enrollment.

### Participants

Women who were 18 years or older, had engaged in sexual activity in the past six months, and were capable of completing the questionnaires were considered eligible. Exclusion criteria were: severe psychiatric disorders (eg, schizophrenia, bipolar disorder, major depressive disorder); major endocrine disorders (eg, diabetes mellitus, Cushing’s syndrome, hyperthyroidism); pregnancy, lactation, or postpartum status within the last 6 months; history of thyroid, breast, or pituitary tumors; initiation of thyroid-related medication within the last 3 months; severe chronic systemic disease (eg, renal/hepatic failure); active alcohol or substance use disorder; and history of sexual trauma within the past year.

Participants using medications known to affect sexual function or PRL levels, including antidepressants, antipsychotics, hormonal contraceptives, hormone replacement therapy, dopamine-modulating agents, and flibanserin, were excluded. No participant reported current use of flibanserin.

### Prolactin

Fasting venous blood samples were collected between 08:00 and 10:00 AM. Serum PRL levels were measured using high-sensitivity immunoassay methods on an Abbott ARCHITECT i2000SR immunoassay autoanalyzer (Abbott Laboratories, Abbott Park, IL, United States) at the hospital laboratory.

Blood samples were obtained during routine outpatient evaluation. Although sampling was standardized by morning timing and fasting status, macroprolactin screening was not routinely performed, and blood sampling was not standardized according to menstrual cycle phase.

Participants were categorized as having normal PRL (≤25 ng/mL) or elevated PRL (>25 ng/mL) according to the clinical reference threshold used by the study laboratory. This dichotomous grouping was applied in comparative and multivariable analyses.

### Measures

A structured form captured age, marital status, education, income, smoking status, alcohol use, comorbid medical illness, and psychological illness. Relationship-related variables included current sexual partnership status, relationship duration, and history of premarital relationships.

Sexual knowledge was assessed using a four-item index developed for the present study. Items evaluated factual knowledge regarding (1) external genital anatomy, (2) clitoral structure and function, (3) vaginal anatomy and physiological response, and (4) prior sexual health education exposure. Items were scored on ordinal scales reflecting accuracy of responses, with higher scores indicating greater knowledge. A composite Sexual Knowledge score was calculated as the mean of the 4 items and treated as a continuous variable in analyses. Internal consistency in the present sample was acceptable (Cronbach’s α = 0.70). Corrected item–total correlations ranged from 0.36 to 0.59. As the index was developed for this study, it should be considered exploratory and requires further validation.

Sexual behavior variables included pornography use (yes/no), knowledge of Kegel exercise (yes/no), and practice of Kegel exercise (yes/no).

Sexual distress was assessed with the 13-item FSDS-R, which evaluates sexually related personal distress over the past 30 days. Total score ranges from 0 to 52, and higher scores indicate greater distress.^19^ The validated Turkish version was used; internal consistency in the present sample was high (Cronbach’s α = 0.87–0.99).^20^ The FSDS-R total score was analyzed as a continuous outcome.

### Statistical analysis

Statistical analyses were performed using IBM SPSS Statistics for Windows, Version 27.0 (IBM Corp., Armonk, NY, United States).

An a priori power calculation was performed using G*Power, Version 3.1.9.7. For the primary analysis of covariance (ANCOVA) comparison evaluating the PRL group effect (df = 1), approximately 199 participants were required to achieve 80% power to detect a small-to-moderate effect size (Cohen’s f = 0.20) at α = 0.05. The final sample (*N* = 200) exceeded this requirement.

Continuous variables are presented as mean ± standard deviation (SD), and categorical variables as frequency (percentage). Normality of continuous variables was assessed using visual inspection of histograms and Shapiro–Wilk tests. Homogeneity of variance was evaluated using Levene’s test. There was no missing data for primary outcome variables; analyses were conducted on complete cases. Assumptions for ANCOVA, including homogeneity of regression slopes, were examined prior to model estimation. Between-group comparisons (PRL ≤25 vs >25 ng/mL) used independent-samples t-tests for continuous variables and chi-square tests for categorical variables. The independent association between elevated PRL and sexual distress was examined using ANCOVA with FSDS-R total scores as the dependent variable. Model 1 included PRL group as the main predictor and adjusted for age, comorbid medical illness, and psychological illness. Model 2 additionally included the Sexual Knowledge Index, Kegel exercise knowledge, and Kegel exercise practice. Estimated marginal means (±SE) were reported for PRL groups. Effect sizes were summarized as partial eta squared (η^2^). Two-sided *P* values <0.05 were considered statistically significant. As a secondary exploratory analysis, serum PRL was stratified into sample-based quartiles using the 25th, 50th, and 75th percentile cutoffs: Q1 ≤ 11.375 ng/mL, Q2 11.376–17.965 ng/mL, Q3 17.966–27.090 ng/mL, and Q4 > 27.090 ng/mL. PRL quartile was first evaluated in relation to FSDS-R total score using the Kruskal–Wallis test, followed by Bonferroni-adjusted pairwise comparisons. PRL quartile was then entered as a 4-level fixed factor in ANCOVA models using the same covariate sets as Models 1 and 2. Estimated marginal means (±SE) were calculated for each quartile, and pairwise comparisons were adjusted using the Bonferroni correction. These analyses were considered exploratory and were performed to evaluate potential graded or non-linear associations that may have been obscured by dichotomization.

## Results

A total of 200 women were included (mean age 35 ± 8 years). Sixty-five participants (32.5%) had elevated PRL (>25 ng/mL), and 135 (67.5%) had PRL ≤25 ng/mL. Baseline group comparisons are presented in Table 1.

**Table 1:** Baseline characteristics by PRL status.

Sociodemographic characteristics	Total sample (*N* = 200)	PRL ≤ 25 ng/mL (*n* = 135)	PRL > 25 ng/mL (*n* = 65)	T/× ^2^	*P*
Age, years	35 **±** 8	34.6 ± 7.6	35.7 ± 8.5	−0.913	.363
Marital status				0.890	.345
Married	189 (94.5)	129 (95.6)	60 (92.3)		
Single	11 (5.5)	6 (4.4)	5 (7.7)		
Education level				0.509	.917
Primary School	38 (19)	26 (19.3)	13 (20.0)		
Middle School	68 (34)	44 (32.6)	24 (36.9)		
High School	59 (29.5)	41 (30.4)	18 (27.7)		
University	34 (17)	24 (17.8)	10 (15.4)		
Income level				2.010	.366
Low	122 (61.3)	81 (60.0)	41 (63.1)		
Middle	59 (29.6)	43 (31.9)	16 (24.6)		
High	18 (9)	10 (7.4)	8 (12.3)		
**Clinical/hormonal** **variables**					
PRL, ng/mL	17.9 (15.5)				
PRL >25 ng/mL	65 (32.5)	−	−	−	−
Comorbid medical illness	44 (22.1)	29 (21.5)	15 (23.1)	0.096	.756
Medication use	37 (18.6)	22 (16.3)	15 (23.1)	1.282	.257
Psychological illness	27 (13.5)	16 (11.9)	11 (16.9)	0.927	.336
History of sexual trauma	9 (4.5)	6 (4.4)	3 (4.6)	0.003	.956
**Lifestyle factors**					
Current smoker	55 (27.5)	34 (25.2)	21 (32.3)	3.845	.146
Alcohol use	18 (9)	10 (7.4)	8 (12.3)	1.318	.517
**Relationship context**					
Sexual partner present	152 (76)	104 (77.0)	48 (73.8)	0.245	.621
Duration of relationship (years)	14.8 (**±** 9.3)	14.5 ± 9.1	15.3 ± 9.9	0.482	.629
Premarital relationship	94 (47.2)	62 (45.9)	32 (49.2)	0.154	.695
Duration of premarital relationships (years)	2.4 **±** 2.8	2.2 ± 2.5	2.8 ± 3.2	1.186	.236

Medication use refers only to medications that did not meet exclusion criteria and were not considered likely to affect prolactin levels or sexual function. Data are presented as mean ± SD or *n* (%), as appropriate. Group comparisons were performed using the independent-samples *t* test for continuous variables and the chi-square test (or Fisher’s exact test when appropriate) for categorical variables. PRL groups were defined as ≤25 ng/mL vs >25 ng/mL. Abbreviations: PRL = prolactin; SD = standard deviation.

Sexual knowledge and sexual health behaviors were similar across PRL groups. The composite Sexual Knowledge Index did not differ by PRL status (*P* = .203), and levels of knowledge about external genital anatomy, clitoral function, vaginal structure/function, and sexual health education were comparable (all *P* > .05). Pornography use, Kegel exercise knowledge, and Kegel exercise practice did not differ significantly between groups (all *P* > .05). Despite similar knowledge/behavior profiles, women with elevated PRL had higher sexual distress scores (FSDS-R total score 26.1 ± 12.5 vs 22.2 ± 9.4; *P* = .029) (Table 2).

**Table 2:** Sexual knowledge, behavioral variables, and distress by PRL status.

Sexual knowledge and behavioral variables	Total sample (*N* = 200)	PRL ≤ 25 ng/mL (*n* = 135)	PRL > 25 ng/mL (*n* = 65)	t/χ ^2^ /z	*P*
Sexual Knowledge Index	1.79 ± 0.57	1.75 (0.75)	1.75 (1.00)	1.274	.203
Knowledge of external genital anatomy				1.940	.585
No knowledge	57 (28.5)	41 (30.4)	16 (24.6)		
Limited	65 (32.5)	43 (31.9)	22 (33.8)		
Moderate	61 (30.5)	38 (28.1)	23 (35.4)		
Good	17 (8.5)	13 (9.6)	4 (6.2)		
Knowledge of clitoral function				2.785	.248
No knowledge	124 (62)	88 (65.2)	36 (55.4)		
Partial	42 (21)	28 (20.7)	14 (21.5)		
Full	34 (17)	19 (14.1)	15 (23.1)		
Knowledge of vaginal structure/function				1.108	.575
No knowledge	60 (30)	43 (31.9)	17 (26.2)		
Partial	101 (50.5)	68 (50.4)	33 (50.8)		
Full	39 (19.5)	24 (17.8)	15 (23.1)		
Sexual health education				2.246	.325
No education	118 (59)	83 (61.5)	35 (53.8)		
Self-learning	60 (30)	36 (26.7)	24 (36.9)		
Formal education	22 (11)	16 (11.9)	6 (9.2)		
Sex toy use	4 (2)	3 (2.2)	1 (1.5)	0.105	.608
Pornography use	51 (25.5)	29 (21.5)	22 (33.8)	3.531	.060
Kegel exercise knowledge	62 (31)	37 (27.4)	25 (38.5)	2.506	.113
Kegel exercise practice	28 (14)	17 (12.6)	11 (16.9)	0.683	.408
**Sexual distress score**					
FSDS-R total score	23.5 ± 10.7	22.2 ± 9.4	26.1 ± 12.5	-2.212	**.029**

Data are presented as mean ± SD, median (IQR), or *n* (%), as appropriate. Group comparisons were performed using the independent-samples t test for normally distributed continuous variables, the Mann–Whitney *U* test for non-normally distributed continuous variables, and the chi-square test or Fisher’s exact test for categorical variables, as appropriate. For non-normally distributed continuous variables, the reported *z* value represents the standardized Mann–Whitney *U* test statistic. Bold values indicate statistically significant results at *p* < 0.05. Abbreviations: PRL = prolactin; FSDS-R = Female Sexual Distress Scale–Revised; SD = standard deviation; IQR = interquartile range.

In ANCOVA Model 1 (adjusted for age, comorbidity, and psychological illness), elevated PRL was significantly associated with higher FSDS-R total scores (F = 5.396, *P* = .021, partial η^2^ = 0.027). Adjusted mean FSDS-R total scores were 27.48 (SE = 1.56) in the elevated PRL group and 23.76 (SE = 1.31) in the normal PRL group.

In Model 2, which additionally adjusted for Sexual Knowledge Index, Kegel exercise knowledge, and Kegel exercise practice, elevated PRL remained significantly associated with greater sexual distress (F = 5.169, *P* = .024, partial η^2^ = 0.026). Higher sexual knowledge was independently associated with lower FSDS-R total scores (F = 4.319, *P* = .039, partial η^2^ = 0.022), whereas Kegel-related variables were not significant (*P* > .05). Adjusted mean FSDS-R total scores were 28.23 (SE = 1.76) for elevated PRL and 24.58 (SE = 1.58) for normal PRL (Table 3).

**Table 3:** ANCOVA models predicting FSDS-R total score.

	Model 1			Model 2		
Predictor	F	*P*	Partial η^2^	F	*P*	Partial η^2^
Age	0.264	.608	0.001	0.208	.649	0.001
Comorbidity	1.354	.246	0.007	1.208	.273	0.006
Psychological illness	1.162	.282	0.006	1.858	.175	0.010
PRL > 25 ng/mL	**5.396**	**.021**	**0.027**	**5.169**	**.024**	**0.026**
Sexual knowledge	−	−	−	**4.319**	**.039**	**0.022**
Kegel exercise practice	−	−	−	0.000	.996	0.000
Kegel exercise knowledge	−	−	−	2.660	.105	0.014

Analysis of covariance (ANCOVA) models predicting FSDS-R total score are shown. Partial η^2^ indicates effect size. Bold values indicate statistically significant results at *p* < 0.05. Model 1 adjusted for age, comorbidity, and psychological illness; Model 2 additionally adjusted for Sexual Knowledge Index, Kegel exercise knowledge, and Kegel exercise practice. Abbreviations: PRL = prolactin; FSDS-R = Female Sexual Distress Scale–Revised.

An exploratory analysis testing the interaction between PRL status and Sexual Knowledge did not demonstrate a significant interaction effect (F = 0.16, *P*  = .69, partial η^2^ = 0.001). The absence of a significant interaction indicates that sexual knowledge did not significantly moderate the association between PRL status and FSDS-R total score. However, as shown in Model 2, the Sexual Knowledge Index remained independently associated with FSDS-R total score, suggesting an independent rather than moderating association with sexual distress.

As a secondary exploratory analysis, serum PRL was categorized into sample-based quartiles. FSDS-R total scores differed significantly across PRL quartiles in the unadjusted analysis (Kruskal–Wallis H[3] = 8.833, *P* = .032), with Bonferroni-adjusted pairwise comparisons showing a significant difference only between Q2 and Q4 (adjusted *P* = .019). PRL quartile also remained significantly associated with FSDS-R total scores in both adjusted ANCOVA models (Model 1: F[3192] = 4.394, *P* = .005, partial η^2^ = 0.064; Model 2: F[3189] = 4.579, *P* = .004, partial η^2^ = 0.068). In both adjusted models, the only Bonferroni-corrected pairwise comparison that remained significant was Q2 versus Q4. The quartile analysis suggested a non-monotonic pattern, with the highest distress scores observed in the upper PRL quartile and no evidence that the lowest PRL quartile was associated with higher sexual distress (Table 4).

**Table 4:** Exploratory analysis of FSDS-R total score across serum PRL quartiles.

PRL quartile	PRL range, ng/mL	*n*	Unadjusted FSDS-R total, mean ± SD	Model 1 adjusted mean ± SE	Model 2 adjusted mean ± SE
Q1	≤11.375	50	23.68 ± 9.92	23.58 ± 1.48	23.78 ± 1.47
Q2	11.376–17.965	50	19.66 ± 6.43	19.71 ± 1.50	19.42 ± 1.49
Q3	17.966–27.090	50	23.40 ± 10.55	23.33 ± 1.48	23.60 ± 1.49
Q4	>27.090	50	27.36 ± 13.52	27.33 ± 1.47	27.14 ± 1.48

Data are presented as mean ± SD for unadjusted FSDS-R total scores and estimated marginal mean ± SE for adjusted models. PRL quartiles were defined using sample-based 25th, 50th, and 75th percentile cutoffs. The unadjusted comparison was performed using the Kruskal–Wallis test and showed a significant overall difference across quartiles (H[3] = 8.833, *P* = .032); Bonferroni-adjusted pairwise comparisons showed a significant difference only between Q2 and Q4 (adjusted *P* = .019). Model 1 was adjusted for age, comorbid medical illness, and psychological illness (F[3192] = 4.394, *P* = .005, partial η^2^ = 0.064). Model 2 was additionally adjusted for Sexual Knowledge Index, Kegel exercise knowledge, and Kegel exercise practice (F[3189] = 4.579, *P* = .004, partial η^2^ = 0.068). In both adjusted models, only the Q2–Q4 pairwise comparison remained significant after Bonferroni correction.

Abbreviations: PRL = prolactin; FSDS-R = Female Sexual Distress Scale–Revised; SD = standard deviation; SE = standard error.

## Discussion

In this cross-sectional study involving women visiting a gynecology outpatient clinic, elevated serum PRL levels were found to be independently associated with higher sexual distress, after adjustment for age, comorbid medical illness, and psychological illness. The association remained significant after further controlling sexual knowledge and Kegel-related behaviors. Additionally, higher sexual knowledge was independently associated with lower levels of sexual distress. These findings support a biopsychosocial model in which neuroendocrine and cognitive factors independently contribute to women’s subjective sexual well-being.

Previous research has primarily focused on sexual function outcomes rather than sexual distress specifically. Although overt hyperprolactinemia is a recognized contributor to sexual dysfunction,^12^^,^^13^^,^^15^ studies examining distress specifically using validated measures such as the FSDS-R remain limited. In retrospective clinical samples, hyperprolactinemic women constituted small subgroups and showed higher FSDS-R total scores than normoprolactinemic women, but limited sample sizes constrained precision and generalizability.^15^^,^^21^ Our results suggest that PRL status may be relevant not only to physiological aspects of sexual response but also to the subjective appraisal and emotional burden associated with sexual experiences. Importantly, the observed effect sizes were modest, implying that PRL is one of many factors in a complex biopsychosocial framework rather than a primary cause of distress. Nonetheless, even minor independent effects could be clinically significant in multifaceted conditions like female sexual dysfunction.

To evaluate whether dichotomization of PRL obscured potentially relevant associations, we performed a secondary exploratory analysis stratifying PRL into sample-based quartiles. PRL quartile was significantly associated with FSDS-R total scores in both unadjusted and adjusted analyses. The observed pattern was non-monotonic rather than strictly linear: Q2 showed the lowest distress scores, whereas Q4 showed the highest distress scores. The only Bonferroni-corrected pairwise contrast that remained significant was Q2 versus Q4.

This finding is consistent with prior evidence that hyperprolactinemia is associated with impaired female sexual function, including reduced desire and dysfunction across multiple sexual function domains. However, the quartile findings also suggest that the relationship between PRL and female sexual outcomes may not be fully captured by a simple linear model. Previous work has also suggested that low PRL levels may be related to hypoactive sexual desire disorder and altered sexual inhibition profiles, indicating that PRL may have different associations depending on the specific sexual outcome assessed. In the present study, however, the lowest PRL quartile did not show significantly higher sexual distress than the other quartiles. Therefore, while our findings support the relevance of higher PRL levels, particularly in the upper quartile, they do not provide robust evidence that lower PRL levels are independently associated with worse sexual distress.

Sexual knowledge was evaluated as a secondary exploratory cognitive-educational variable within the biopsychosocial framework of female sexual distress. This variable was included because knowledge of genital anatomy, sexual response, and sexual health may shape how women interpret sexual experiences and may reduce distress through fewer misconceptions and greater sexual self-efficacy. In the fully adjusted model, higher sexual knowledge was independently associated with lower distress, even though it did not significantly moderate the association between PRL status and FSDS-R total score. This suggests that biological and cognitive-educational factors may contribute independently, rather than interactively, to women’s subjective sexual distress. This interpretation is consistent with cross-sectional structural equation modeling data, which show that sexual health literacy has both direct effects on sexual function and indirect effects via sexual satisfaction, with self-efficacy independently contributing to better outcomes.^22^ Similarly, intervention studies among breast and cervical cancer survivors indicate that structured sexual education and counseling programs improve sexual function and reduce sexual distress. Together, these findings support the view that cognitive and educational dimensions are not merely correlates but potentially modifiable contributors to sexual distress.^23^^–^^25^

Kegel exercise knowledge and practice were included as secondary exploratory behavioral variables because pelvic floor-related behaviors may influence sexual function and may indirectly affect sexual distress. However, these variables were not independently associated with distress in the multivariable model. The dichotomous measurement of these variables likely limited sensitivity, as frequency, supervision, technique quality, and clinical indication were not assessed. Although supervised pelvic floor muscle training (PFMT) has been shown to improve multiple FSFI domains and reduce sexual distress compared with control conditions,^26^ meta-analyses indicate substantial heterogeneity and generally low-to-moderate quality evidence.^27–29^ Moreover, improvements in specific sexual function domains do not uniformly translate into reductions in global sexual distress. Our findings, therefore, underscore the distinction between functional improvement and subjective distress.

These findings support a clinical approach that considers both biological and cognitive/educational dimensions. In women presenting with sexual distress, assessing PRL status may be informative, particularly when distress is disproportionate to reported symptom severity. At the same time, structured sexual education may represent a low-risk, accessible intervention that addresses cognitive appraisal processes contributing to distress.

Strengths of this study include the sample size, systematic assessment of key covariates, and use of a validated measure of sexual distress. However, several limitations should be acknowledged. The cross-sectional design precludes causal inference. Reverse causation also cannot be excluded, as psychological or sexual distress may itself influence PRL secretion through stress-related neuroendocrine mechanisms, particularly in the context of mild PRL elevations.

PRL was measured at a single time point, and physiological fluctuations were not assessed. In addition, because blood samples were obtained during routine outpatient evaluation, macroprolactin screening was not routinely performed, and sampling was not standardized according to menstrual cycle phase. These factors may have influenced the interpretation of mild PRL elevations and should be addressed in future studies with repeated hormone measurements and more detailed endocrine characterization.

The Sexual Knowledge Index was developed for this study and requires external validation. The sample was clinic-based and may not generalize to community populations. Residual confounding, including relationship quality, partner factors, depressive symptom severity, and other hormonal variables, cannot be excluded. All psychosocial measures relied on self-report.

Future longitudinal studies should examine whether normalization of elevated PRL leads to measurable reductions in sexual distress and whether psychoeducational interventions independently reduce distress across hormonal strata. Validation of multidimensional sexual knowledge instruments and incorporation of relational and affective mediators would further clarify mechanisms linking biological and cognitive domains to sexual distress.

## Conclusion

Elevated PRL levels are independently associated with higher sexual distress, while greater sexual knowledge is independently associated with lower distress. These findings support a biopsychosocial framework in which hormonal and cognitive factors contribute additively to women’s subjective sexual well-being. Integrated medical and educational strategies may therefore be beneficial in the evaluation and management of female sexual distress.

## Data Availability

The data supporting the findings of this study are available from the corresponding author upon reasonable request.

## Preprint and presentation statement

This manuscript has not been posted as a preprint and has not been presented at a scientific meeting.
